# Safety evaluation of *Weissella confusa* SY628 and the effect of its fermentation on the taste and quality of soy yogurt

**DOI:** 10.3389/fmicb.2025.1567399

**Published:** 2025-05-14

**Authors:** Muwen Liu, Xiao Li, Tao Ye, Liangzhong Zhao, Xuejiao Zhang

**Affiliations:** Hunan Provincial Key Laboratory of Soybean Products Processing and Safety Control, Shaoyang University, Shaoyang, China

**Keywords:** *Weissella confusa* SY628, soy yogurt, starter, physical and chemical properties, flavor quality

## Abstract

In the global context, the demand for sustainable protein sources is rising, spotlighting plant—based foods, especially legume products. Fermentation is crucial in developing these foods, as it reduces anti—nutritional factors and improves flavor. But the available fermentation strains for plant—based foods are limited. This study aims to address this knowledge gap by evaluating the safety of *Weissella confusa* SY628 as a fermentation strain and its impact on the quality characteristics of soy yogurt. The safety evaluation of *W. confusa* SY628 demonstrated that it possessed no hemolytic activity and was sensitive to a variety of antibiotics, no biogenic amines were produced, suggesting an extremely low pathogenic risk. Furthermore, *W. confusa* SY628 demonstrated enhanced acid and bile tolerance, characteristics that are indicative of its probiotic properties. The fermentation of soy yogurt was conducted using three distinct organisms: *W. confusa* SY628, commercial bacterial powder CHS, and a combined starter SYCHS composed of the two aforementioned organisms. The physical, and chemical properties and taste quality of the samples were measured. The results demonstrated that in the SYCHS group, after a 21-day storage period, the pH level was 4.49, the total acidity reached 76.77 °T, and the viable count was 5.81 × 10^7^ CFU/mL, indicating good storage stability. The cohesiveness, viscosity, elasticity, and storage modulus of the SYCHS group were found to be significantly higher than those of the other groups, and the internal network structure was found to be stable. In the SYCHS group, the total amino acid content was determined to be 308.57 μg/g, with umami-tasting amino acids accounting for 22.95%. The total fatty acid content was found to be 1818.95 μg/g, with a notably high polyunsaturated fatty acid content, indicating significant nutritional value. The SYCHS group exhibited the highest number of key flavor components. Substances such as 2,3-butanedione exhibited high ROAV values, contributing to a rich flavor profile. In conclusion, the co-fermentation of *W. confusa* and commercial bacteria significantly improved the overall quality of soy yogurt, providing a theoretical and practical basis for the innovative development of plant-based foods.

## 1 Introduction

The global food industry has undergone significant development, and consumers have demonstrated an increased interest in healthy nutrition. Consequently, plant-based foods have emerged as a focal point of research due to their environmental sustainability, nutritional value, and health benefits (Canoy et al., 2024). Soy yogurt is classified as a type of plant-based fermented food product. It is distinguished by its low-fat content, absence of cholesterol, and abundance of plant protein. The product under discussion is not only suitable for individuals with lactose intolerance; it has also become a healthy choice for an increasing number of consumers (Zhang et al., 2024). Notwithstanding, soy yogurt continues to encounter difficulties with regard to structural stability, mouthfeel smoothness, and flavor complexity. These deficiencies result in constraints on its market promotion and consumer acceptance (Cao et al., 2019; Zhu et al., 2020).

Fermentation represents a pivotal step in the quality enhancement of soy yogurt, with the selection of starter microorganisms exerting a pivotal influence on the fermentation outcome (do Prado et al., 2022). It has been demonstrated that traditional starter cultures are incapable of enhancing the quality of soy yogurt to a satisfactory degree (Harper et al., 2022). Consequently, the development of novel and highly efficient starter cultures has become a focal point of research endeavors (Li C. C. et al., 2024; Li S. et al., 2024). *Weissella confusa*, a species of lactic acid bacterium commonly found in various fermented foods, has seen a surge in utilization within the food industry due to its distinctive fermentation characteristics and biological functions (Sharma et al., 2018; Wan et al., 2023). *W. confusa* possesses notable acid, salt, and heat tolerance, in addition to the capacity to produce a range of beneficial metabolites during the fermentation process. These include organic acids, esters, and amino acids, which play a pivotal role in enhancing the flavor and nutritional value of foods (Zhu et al., 2018). During the fermentation process of soy yogurt, *W. confusa*, functioning as an auxiliary fermentation strain, has been shown to enhance the structural properties of the yogurt, contributing to a layered taste profile. Additionally, it has been demonstrated to improve the flavor of soy yogurt through its capacity to produce substances that enhance flavor. Consequently, soy yogurt approaches a level of similarity with traditional dairy yogurt in terms of both taste and flavor (Lorusso et al., 2018). Furthermore, certain natural products produced by *W. confusa* during the fermentation process, such as polysaccharides, have demonstrated potential health benefits. These include promoting gastrointestinal health and enhancing immune function. The presence of these functional ingredients contributes to the enhanced health-promoting properties of soy yogurt.

Despite the numerous benefits demonstrated by the use of *W. confusa* in the fermentation of soy yogurt, its mechanism of action remains incompletely understood, and its use in actual production is not yet widespread (Singh et al., 2024). Consequently, conducting in-depth research on the mechanism by which *W. confusa*, as an auxiliary fermentation strain, affects the structure and flavor of soy yogurt, and optimizing the fermentation process, holds significant theoretical value and potential practical applications for enhancing the quality of soy yogurt and promoting the development of the plant-based food industry (Saffarionpour, 2024). The objective of this study is to methodically examine the function of *W. confusa* in the fermentation process of soy yogurt. The primary objective is to optimize the effects of *W. confusa* on the structural integrity, tactile texture, and gustatory profile of yogurt. Additionally, the study seeks to elucidate its potential in plant-based fermented foods and to furnish both theoretical substantiation and practical guidance for the industrial production of soy yogurt. The study’s findings are expected to generate novel concepts for research and applications in the domain of plant-based foods.

## 2 Materials and methods

### 2.1 Raw materials

Soybeans and sucrose were purchased from the local market (Shaoyang, China). *Weissella confuse* SY628: The deposit number is CGMCC No.32006 (deposited at the China General Microbiological Culture Collection Center). *Lactobacillus rhamnosus* (LGG, GDMCC 1.1798) was purchased from the Guangdong Institute of Microbiology. Direct-vat-set commercial starter: CHS (a mixed inoculum of *Lactobacillus delbrueckii* subsp. bulgaricus and *Streptococcus thermophilus* at a ratio of 1:1, produced by Chr. Hansen A/S, Denmark). All other chemicals used were analytical grade and commercially available.

### 2.2 Strains and growth conditions

The activated *W. confusa* SY628 and *Lactobacillus rhamnosus* were each inoculated into 100 mL of MRS medium at 2% (v/v) and then cultured at 37°C for 16 h to obtain the seed liquid for fermentation (Jang et al., 2021). Centrifuge the Seed Liquid at 5000 rpm for 5 min at 4°C. Wash three times with sterile physiological saline and adjust to a concentration of 1 × 10^8^ CFU/mL to serve as the seed broth for fermentation.

### 2.3 Evaluation of the probiotic properties of the bacterial strains

According to the method of Rahman et al. (2024) and Rahmati-Joneidabad et al. (2024), with slight modifications of the strains were evaluated with slight modifications.

#### 2.3.1 Acid tolerance

The pH of the MRS broth medium was adjusted to 1.5, 2.5, 3.5, 4.5, and 5.5. *W. confuse* 628 and LGG were inoculated at 2% (v/v) each, and after incubation at 37°C for 24 h, dilution spread plating was performed on MRS plates for colony counting.

#### 2.3.2 Bile salt tolerance

The activated seed liquid was inoculated at 1% in a sterilized MRS liquid medium containing different concentrations (0.05–1.5% w/v) of bile salts (prepared by mixing bile salts with MRS broth solution). After incubation at 37°C for 24 h, dilution spread plating was performed on MRS plates for colony counting.

#### 2.3.3 Artificial gastrointestinal fluid tolerance

Take 1.0 mL (9 log CFU/mL) of the activated strain solution and mix it with 9 mL of simulated gastric fluid at pH = 3. Incubate at 37°C with shaking at 100 rpm for 1 h, 2 h, and 3 h, respectively. Also, mix with 1.0 mL simulated intestinal fluid containing 1 g/100 mL trypsin at pH = 8 and incubate at 37°C for 0 h, 2 h, and 4 h. Then centrifuge all samples at 5000 × g for 10 min, discard the supernatant, collect the precipitate and count CFU/mL on MRS agar using the standard plate count method.

#### 2.3.4 Antibacterial activity

Using Escherichia coli and Staphylococcus aureus as indicator bacteria, incubate at 37°C for 48 h, then centrifuge at 8000 rpm for 10 min. Filter the supernatant through a 0.22 μm filter under aseptic conditions. Add 100 μL of the indicator bacteria solution to an Oxford cup (h = 7.8 mm) containing LAB solid medium. Allow to diffuse for 5 h, incubate at 37°C for 24 h and measure the diameter of the inhibition zone (mm).

### 2.4 Evaluation of the strain safety characteristics

#### 2.4.1 Hemolysis test

Using Qi et al. (2024) method with slight modifications, streak the strain onto a Columbia Blood Agar plate. Simultaneously, LGG was streaked on the same plate as a negative control. Place these plates in an incubator at 35°C for 48 h and observe for a hemolytic zone around the colonies.

#### 2.4.2 Biogenic amine production

Tyrosine (free-base), histidine monohydrochloride, ornithine monohydrochloride, and lysine monohydrochloride (Macklin Biochemical Co., Ltd., Shanghai, China) were employed as precursor amino acids for biogenic amine (BA) synthesis. The BA-producing capacity of the strain SY628 was evaluated according to the methodology described by Liu M. et al. (2024) and Liu Y. et al. (2024). The strain SY628 were streaked onto the media and incubated at 35°C under constant temperature for 4 days. Colonies were subsequently examined for the presence of purple halos surrounding the bacterial growth, with amino acid-deficient media serving as negative controls. The experiments were conducted in triplicate.

#### 2.4.3 Antibiotic susceptibility testing

Following the method of Jaqueline (Almeida et al., 2024), the sensitivity of *W. confuse* 628 and LGG to 8 types of antibiotics on MRS agar was determined using the standard paper disk diffusion method. The samples were incubated under facultative anaerobic conditions at 35°C for 24 h. The antibiotic discs used contained amikacin (30 μg), erythromycin (15 μg), penicillin (10 U/μg), chloramphenicol (30 μg), cefazolin (30 μg), ampicillin (10 μg), co-trimoxazole (23.75 μg) and amoxicillin (20/10 μg).

### 2.5 Sample preparation

The method was slightly adjusted according to the existing formulation and process in the laboratory.

1 kg of soybeans was weighed, and soaked in cold water at a soybean-to-water ratio of 1:4 for 8–10 h, then mix them with water at a soybean-to-water ratio of 1:8 (w/w, based on dry soybeans) and grind the mixture using a two-stage tandem high-concentration fine grinder (JM—F80, Shanghai Dingpai Machinery Equipment Co., Ltd., China). Boil the soybean milk with 102°C steam for 15 min and filter through a 200-mesh vibrating sieve. Add 6% sucrose for formulation, sterilize at 85°C for 20 min, cool to 34°C, inoculate with 2% (v/v) starter culture, ferment at 37°C for 8 h, and ripen at 4°C for 12 h. The finished product was stored in a refrigerator at 4°C. According to the different fermentation strains added, the samples are named SY628 (1 × 10^8^ CFU/mL *W. confusa* SY628, 2% v/v), CHS (2 g commercial bacterial powder dissolved in 98 g sterile water, 2% v/v) and SYCHS (SY628: CHS = 1:1,2% v/v). All tests were performed in triplicate.

### 2.6 Physicochemical analysis

The total number of colonies: Following Bao et al. (2025), with some modifications, the number of colonies in soy yogurt was determined on days 1, 7, 14, and 21.

#### 2.6.1 Titratable acid

Prepare the sample by diluting (10 g) with distilled water (20 mL). Add phenolphthalein solution (2 mL, 5% w/v) to the diluent as an indicator. Titrate the mixture with 0.1 M sodium hydroxide to a pink color that does not fade within 30 s. The TAA (T) is calculated as the volume (mL) of sodium hydroxide consumed multiplied by 10.

#### 2.6.2 pH

After bringing the sample to room temperature, stir the sample evenly with a glass rod. Measure the pH of the fermented milk in the soy yogurt using a pH meter and repeat the measurement three times for the sample.

### 2.7 Texture testing

After storing the sample in a refrigerator at 4°C for 24 h, remove the sample and allow it to stand at room temperature for 15 min. Use the TPA (Texture Profile Analysis) mode of a texture analyzer (TX—700, CS Scientific, United States) with a TMS 38.1 mm Plexiglas cylindrical plastic probe. Under the conditions of a trigger force of 1 gf, a compression degree of 20%, a pre-test speed of 2 mm/s, and a test speed of 1 mm/s, repeatedly measure the soy yogurt samples stored for 1, 7, 14, and 21 days to obtain texture characteristic data such as hardness, elasticity, cohesiveness, adhesiveness, springiness, gumminess, and chewiness (Ziarno et al., 2023).

### 2.8 Rheological determination

Following the previous research (Abdeldaiem et al., 2023), the rheological properties of the samples were characterized. The soy yogurt samples stored for 1, 7, 14 and 21 days were measured using a rheometer (MCR 302, Anton Paar, Austria). An amplitude sweep was performed on the samples using a parallel plate configuration with a diameter of 40 mm and a gap between the plates of 1 mm.

### 2.9 Color difference detection and sensory evaluation

The chromaticity values L, a, and b of the three samples were measured using a fully automatic color difference meter (Ci7800, Shenzhen Sanshi Technology Co., Ltd., China). Among them, the chromaticity value L represents the brightness. The larger the chromaticity value L, the whiter and brighter the sample appears, while the smaller the L value, the darker and more blackened the sample becomes. The chromaticity value a represents the red-green hue, where a positive value indicates a reddish hue and a negative value indicates a greenish hue. The chromaticity value b represents the yellow-blue hue, where a positive value indicates a yellowish tint and a negative value indicates a bluish tint. Total hue difference formula: $\mathrm{\Delta E}=\sqrt{{\left(\mathrm{\Delta L}\right)}^{2}+{\left(\mathrm{\Delta a}\right)}^{2}+{\left(\mathrm{\Delta b}\right)}^{2.}}$

Sensory evaluation was conducted according to the method reported by Ren et al. (2024) with some changes. A panel of 10 well-trained panelists (five males and five females with age of 18–42) from Shaoyang University were invited for the study. Prior to sensory evaluation, all group members were trained at least 2 h a day on the characteristics of soy yogurt and sensory evaluation requirements for five days. The yogurt samples were divided into different plastic tasting cups with a random code (three-digit numbers), and the samples were tasted in random order. Participants were asked to score yogurt samples according to color, odor, texture, flavor, and overall-acceptability using a 9-point hedonic scale (1: dislike extremely, 9: like extremely).

### 2.10 Microstructure

The lyophilized samples were directly adhered to the conductive adhesive. The samples were then sputtered with gold using an Oxford Quorum SC7620 sputter coater for 45 s at a current of 10 mA. The morphology of the samples was then photographed using a Zeiss Sigma 300 scanning electron microscope (Sigma 300, Carl Zeiss AG, Germany). During morphology photography, the acceleration voltage was 3 kV and the detector used was the SE2 secondary electron detector.

### 2.11 Detection of fatty acids and amino acids

Fatty acid detection by chromatographic conditions and mass spectrometry conditions (Beccaria et al., 2018):

Analysis was performed using a combination of a Thermo Trace 1,300 gas chromatograph system (Thermo Fisher Scientific, United States) and a Thermo ISQ 7000 mass spectrometer (Thermo Fisher Scientific, USA). A Thermo TG—FAME capillary column (50 m × 0.25 mm ID×0.20 μm) was used for the gas chromatography portion. Split injection was performed (injection volume 1 μL, split ratio 8:1). The temperature of the injector was set to 250°C. The temperature programming was as follows: hold at 80°C for 1 min, increase to 160°C at a rate of 20°C/min and hold for 1.5 min, then increase to 196°C at a rate of 3°C/min and hold for 8.5 min, and finally increase to 250°C at a rate of 20°C/min and hold for 3 min. Helium was used as the carrier gas at a flow rate of 0.63 mL/min. For the mass spectrometer part, an electron impact ionization (EI) source with selected ion monitoring (SIM) scanning was used. The electron energy was 70 eV.

Amino acid detection chromatography conditions and measurement of mass spectrometry conditions (Thiele et al., 2019):

An ACQUITY UPLC^®^ BEH C18 chromatography column (2.1 × 100 mm, 1.7 μm, Waters, United States) was used. The injection volume was 5 μL and the column temperature was set at 35°C. The mobile phase A was 50% methanol–water (containing 0.1% formic acid), and the mobile phase B was 10% methanol–water (containing 0.1% formic acid). The gradient elution was performed as follows: from 0–6.5 min, 90–70% B; from 6.5–7 min, 70–0% B; from 7–14 min, 0% B; from 14–14.5 min, 0–90% B; from 14.5–17.5 min, 90% B. The flow rate was 0.3 mL/min from 0–8.0 min and 0.4 mL/min from 8.0–17.5 min. Electrospray ionization (ESI) was used in the positive ion mode. The ion source temperature was 500°C, the voltage was 5,500 V, the collision gas was 4 psi, the curtain gas was 40 psi, and both the nebulizer gas and the auxiliary gas were 50 psi. Multiple reaction monitoring (MRM) scanning was used.

### 2.12 Flavor omics detection

As in the previous study (Li H. et al., 2022), a LECO Pegasus BT 4D GC × GC-TOF MS chromatography system (LECO, St. Joseph, MI, United States) was used. It consisted of an Agilent 8890A gas chromatograph (Agilent Technologies, Palo Alto, CA, United States), a two-stage jet modulator, and a split/splitless injection module. The mass spectrometry system was a high-resolution TOF mass spectrometer detector. The separation system consisted of a first-dimension column DB—Heavy Wax (30 m × 250 μm × 0.5 μm) (Agilent, United States) and a second-dimension column Rxi—5Sil MS (2 m × 150 μm × 0.15 μm) (Restek, United States). The carrier gas was high-purity helium at a flow rate of 1.0 mL/min. For the temperature programming of the first-dimension column: the initial temperature was 50°C and held for 2 min, then increased to 230°C at a rate of 5°C/min and held for 5 min. The temperature programming of the second-dimension column was 5°C higher than that of the first-dimension column. The temperature of the modulator was 15°C higher than that of the second-dimension column and the modulation period was 6.0 s. The injector temperature was 250°C. For the LECO Pegasus BT 4D mass spectrometry detector (LECO, St. Joseph, MI, United States), the transfer line and ion source temperatures were both 250°C. The acquisition rate was 200 spectra/s, the electron impact source was 70 eV, the detector voltage was 1960 V, and the mass spectrometry scan range was m/z 35–550.

The formula for ROAV is ROAV = 100 × (OAV/OAV_max_), where OAV = C/OT. Where C is the concentration of volatile compounds in the sample and OT is the odor threshold of that compound in water. The OT data are taken from the literature.

### 2.13 Data processing

Use SPSS version 23 (IBM SPSS Inc., Chicago, IL, United States) to conduct one-way analysis of variance with Tukey’s test at a 95% confidence level. The numerical values are expressed as mean ± standard deviation. Use Origin 2025 software (Origin Lab Corporation, Northampton, MA, United States) and SIMCA 14.1 software (Biometric Software Developer Umetrics, Umeå, Sweden) for plotting and correlation analysis, respectively.

## 3 Results and discussion

### 3.1 Prebiotic characteristics evaluation

**Figure 1:**
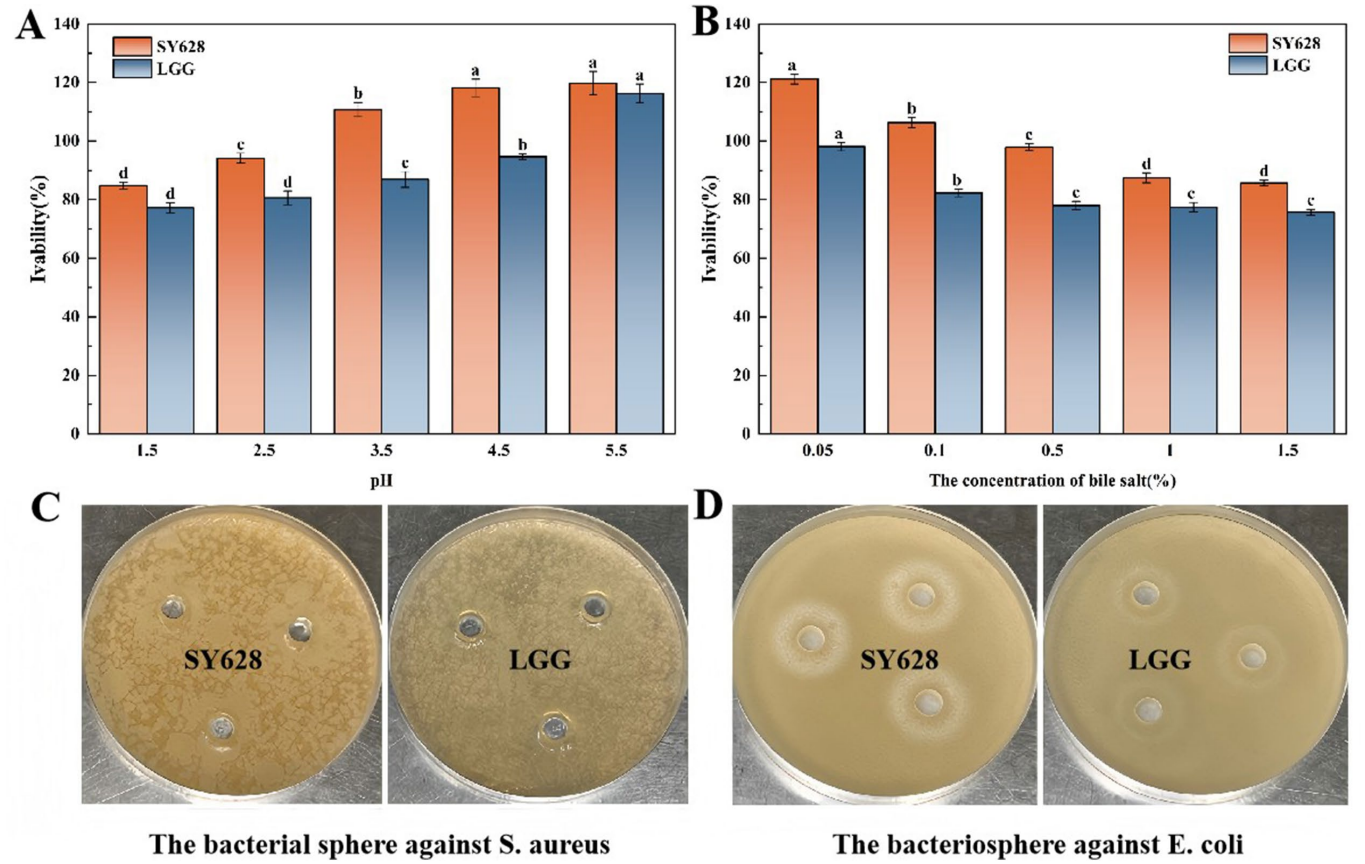
**(A)** Acid tolerance of the strains; **(B)** Bile salt tolerance of the strains; **(C)** Inhibition diagram of *Staphylococcus aureus*; **(D)** Inhibition diagram of *Escherichia coli*.

As shown in Figure 1A, the acid—tolerance experiment demonstrated that the survival rates of SY628 and LGG increase with the rise of pH, and SY628 exhibits better tolerance in a low—pH environment. As depicted in Figure 1B, the viable cell count was measured within the bile—salt concentration range of 0.05–1.5%. With the increase in bile salt concentration, the viable cell counts decreased. The high osmotic pressure outside the cells caused by bile salts affects the bacteria, leading to a decline in the tolerance of the strains (Oberkampf et al., 2022). SY628 shows good bile—salt tolerance. As shown in Figure 1C, neither strain formed a zone of inhibition against *Staphylococcus aureus*. This may be due to the difference in sensitivity of the selected *Staphylococcus aureus* PJ strain compared to previous reports, as well as the specificity of bacteriocin antibacterial activity. Figure 1D shows the antibacterial activity of SY628 and LGG against *Escherichia coli*. The diameter of the inhibition zone of SY628 is 27 ± 0.55 mm, while that of LGG is 23 ± 0.6 mm. SY628 exhibits excellent antibacterial activity against *Escherichia coli*. This could be attributed to the unique physiological properties and metabolites of SY628 that enable it to inhibit *Escherichia coli* more effectively. When used as a fermentation strain, SY628 can effectively reduce the risk of bacterial contamination.

As demonstrated in Table 1, the survival rates of SY628 following treatment with artificial gastric juice and intestinal juice are both higher than those of LGG. After two hours of artificial gastric juice treatment, the survival rate of SY628 is recorded at 70.69% ± 1.23%, while that of LGG is only 40.98% ± 2.31%. After a 3-h treatment period, the survival rate of SY628 remained at 60.15% ± 2.12%, while that of LGG decreased to 27.32% ± 1.65%. In the artificial intestinal juice treatment, SY628 also demonstrates superior performance. The survival rate of SY628 is 68.38% ± 2.75% at 2 h and 29.76% ± 1.86% at 4 h, while the survival rate of LGG is 53.2%. 1% ± 1.94% at 2 h and further decreases to 21.71% ± 1.27% at 4 h. This phenomenon may be attributed to the inherent acid and bile salt tolerance capabilities of *W. confusa*. Its metabolic mechanisms and surface characteristics enable it to withstand the harsh environment of gastric and intestinal juices, thereby enhancing its survival rates (Zhang J. et al., 2023; Zhang X. et al., 2023), It is imperative to maintain a relatively high level of activity. Conversely, LGG exhibits a comparatively diminished capacity to withstand these demands, consequently leading to a reduced survival rate under analogous treatment conditions.

**Table 1:** Artificial gastrointestinal fluid tolerance of the bacterial strains.

Bacterial strain	Simulated gastric fluid		Simulated intestinal fluid	
	Handling time (h)	Fraction surviving (%)	Handling time (h)	Fraction surviving (%)
SY628	2	70.69 ± 1.23^a^	2	68.38 ± 2.75^a^
	3	60.15 ± 2.12^b^	4	29.76 ± 1.86^c^
LGG	2	40.98 ± 2.31^c^	2	53.21 ± 1.94^b^
	3	27.32 ± 1.65^d^	4	21.71 ± 1.27^d^

Different lowercase letters after data in the same column indicate variability.

### 3.2 Safety characteristics of the bacterial strains

**Figure 2:**
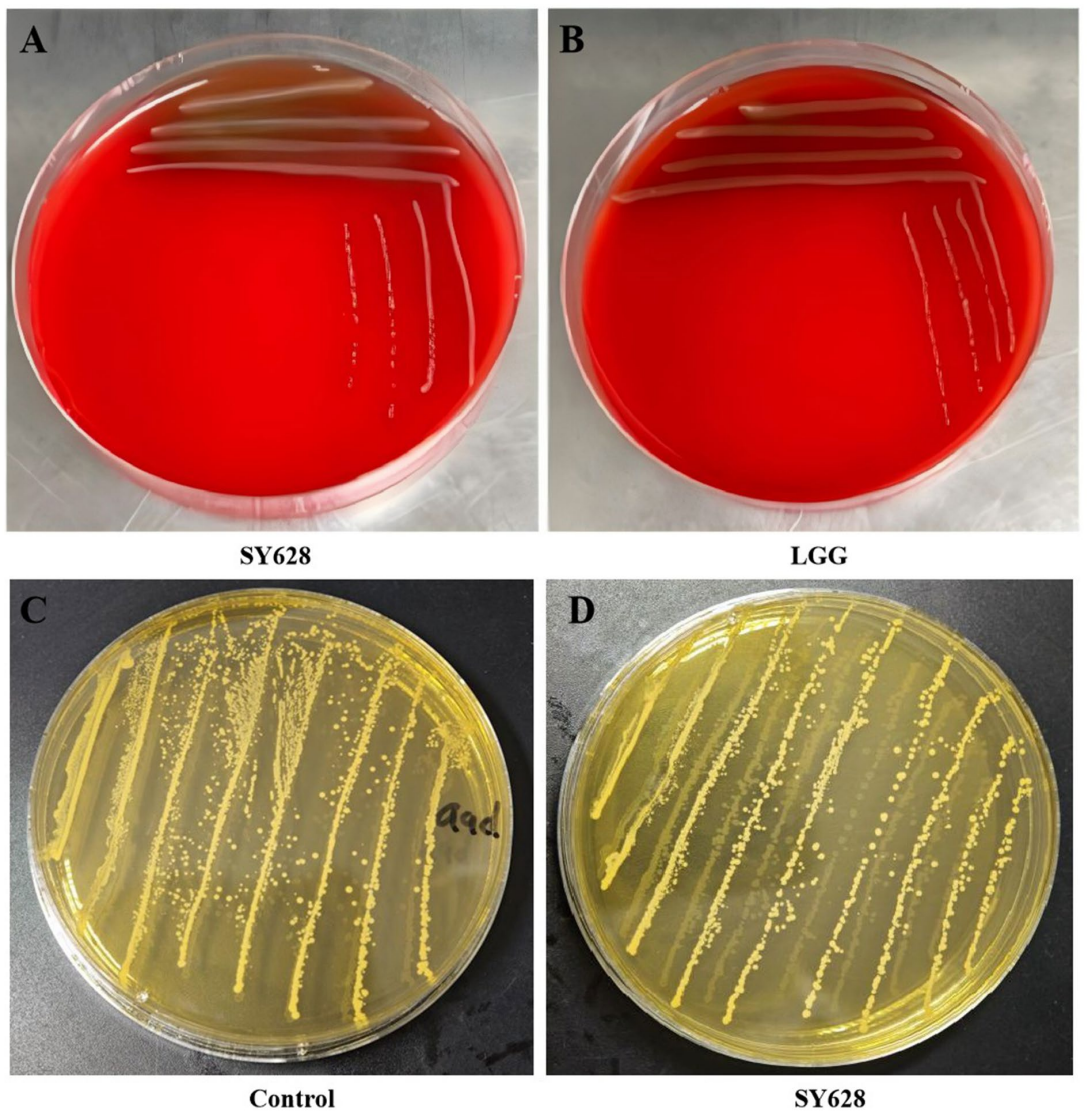
**(A)** SY628 hemolytic activity; **(B)** LGG hemolytic activity; **(C, D)** SY628 bioamine production.

As demonstrated in Table 2, an evaluation of the antibiotic susceptibility of strain SY628 was conducted using eight different antibiotics, with *Lactobacillus rhamnosus* (LGG) serving as the control strain. The results indicated that strain SY628 exhibited resistance to co-trimoxazole and susceptibility to amikacin, erythromycin, chloramphenicol, cefazolin, ampicillin, amoxicillin, aligning with prior research (Liu et al., 2022; Liu M. et al., 2024; Liu Y. et al., 2024). The findings also indicate that penicillin show weak inhibitory effects on strain SY628. LGG exhibited resistance to co-trimoxazole, amikacin, and chloramphenicol. It is noteworthy that resistance to one or more antibiotics is a prevalent characteristic of numerous LAB strains (Rozman et al., 2023). As an LAB strain with potential probiotic properties, SY628 exhibits significantly different antibiotic sensitivities to different antibiotics belonging to the same drug class, suggesting that this strain SY628 may belong to a sensitive isolate. To provide additional information about the safety profile of the test strains, assessments of their hemolytic activity and BA pro-duction were conducted. The results, depicted in Figure 2, indicated that the strain SY628 exhibited no hemolytic activity and BA production, aligning with prior research (Liu M. et al., 2024; Liu Y. et al., 2024) suggesting that SY628 possess safety profile, similar to other lactic acid bacteria.

**Table 2:** Analysis of antibiotic susceptibility of strain SY 628.

Categories of antibiotics	The antibiotic name	SY628 sensitivity	LGG sensitivity
Aminoglycoside	Amikacin	S	R
Macrolides; MIs	Erythromycin	S	S
β-lactam	Penicillin	I	S
Quinolones	Chloramphenicol	S	R
Cephalospora	Cefazolin	S	S
β-lactam	Ampicillin	S	S
Sulfonamide antibiotics	Bactrim	R	R
β-lactam	Amoxicillin	S	S

“S” indicates susceptible, “I” indicates intermediate, and “R” indicates resistant.

### 3.3 Physicochemical analysis

The pH values, total acidity (TA), and viable cell counts of the three samples are shown in Table 3. During storage, the pH of the samples exhibited a gradual decline over time. The SY628 group consistently maintained the highest pH, while the CHS group showed the lowest pH, with the SYCHS group displaying intermediate pH values between the two. This phenomenon may be attributed to the relatively weak lactic acid-producing capacity of SY628, resulting in slower acidity accumulation and minimal pH reduction. In contrast, CHS demonstrated the strongest lactic acid-producing ability, leading to the fastest acidification rate and consequently the lowest pH. For SYCHS, the synergistic interactions between the constituent strains likely modulated the acidification process, balancing the pH reduction rate between those of SY628 and CHS (Maniya et al., 2024). During storage, the total acid content increased continuously and the acidity increased with time. The total acid content of SYCHS was significantly higher than that of SY628 and CHS, indicating that it had the strongest acidifying ability. For SYCHS, the synergistic effect of the three probiotics may lead to the production of more organic acids. Although the acidity of CHS increased rapidly, it leveled off at a later stage, which may be related to the inhibition of end products of lactic acid bacteria metabolism. SY628 had a relatively low acidification capacity, resulting in a slow increase in total acidity (Mohammadi et al., 2017).

**Table 3:** Physicochemical analysis of different samples.

Shelf life		1 d	7 d	14 d	21 d
pH	SY628	5.22 ± 0.03^aA^	5.03 ± 0.02^bA^	4.83 ± 0.03^cA^	4.56 ± 0.03^dA^
	CHS	4.92 ± 0.03^aC^	4.68 ± 0.03^bC^	4.43 ± 0.03^cC^	4.17 ± 0.03^dC^
	SYCHS	5.01 ± 0.03^aB^	4.83 ± 0.02^bB^	4.64 ± 0.02^cB^	4.49 ± 0.03^dB^
ATT/°T	SY628	38.30 ± 0.26^dB^	54.13 ± 0.35^cC^	60.50 ± 0.36^bC^	64.60 ± 0.26^aC^
	CHS	36.23 ± 0.80^dC^	61.37 ± 0.95^cB^	63.70 ± 0.79^bB^	65.97 ± 0.83^aB^
	SYCHS	42.07 ± 0.68^dA^	69.47 ± 0.85^cA^	74.80 ± 0.96^bA^	76.77 ± 0.76^aA^
Mushroom life (10^7^CFU/mL)	SY628	20.50 ± 0.40^bB^	22.04 ± 0.21^aB^	15.95 ± 0.41^cB^	5.10 ± 0.35^dAB^
	CHS	19.37 ± 0.32^bC^	21.21 ± 0.30^aC^	15.55 ± 0.41^cB^	4.42 ± 0.36^dB^
	SYCHS	21.50 ± 0.60^bA^	23.08 ± 0.25^aA^	18.65 ± 0.24^cA^	5.81 ± 0.43^dA^

Lowercase letters represent the differences among various time points during the 21-day storage period of probiotics, while uppercase letters represent the differences among different fermentation agents at the same time point.

For all samples, the viable cell count increased in the early stages of storage (1–7 days) and then gradually decreased. The viable cell count of SYCHS was consistently the highest, while those of CHS and SY628 were lower and decreased more rapidly. SYCHS may be more tolerant and better adapted to the environment and nutrient composition of the soy milk matrix. During the later stages of storage, the activity of lactic acid bacteria was inhibited as the acidity of the soybean yogurt increased. In addition, the reduction of available nutrients in the soy milk matrix during storage limited the proliferation of lactic acid bacteria. These factors resulted in a reduction in the number of viable bacteria (Liang et al., 2022). Analysis of physical and chemical indicators showed that SYCHS had a moderate pH, high acidity, and the highest viable cell count, demonstrating excellent fermentation performance.

### 3.4 Texture profile analysis

**Figure 3:**
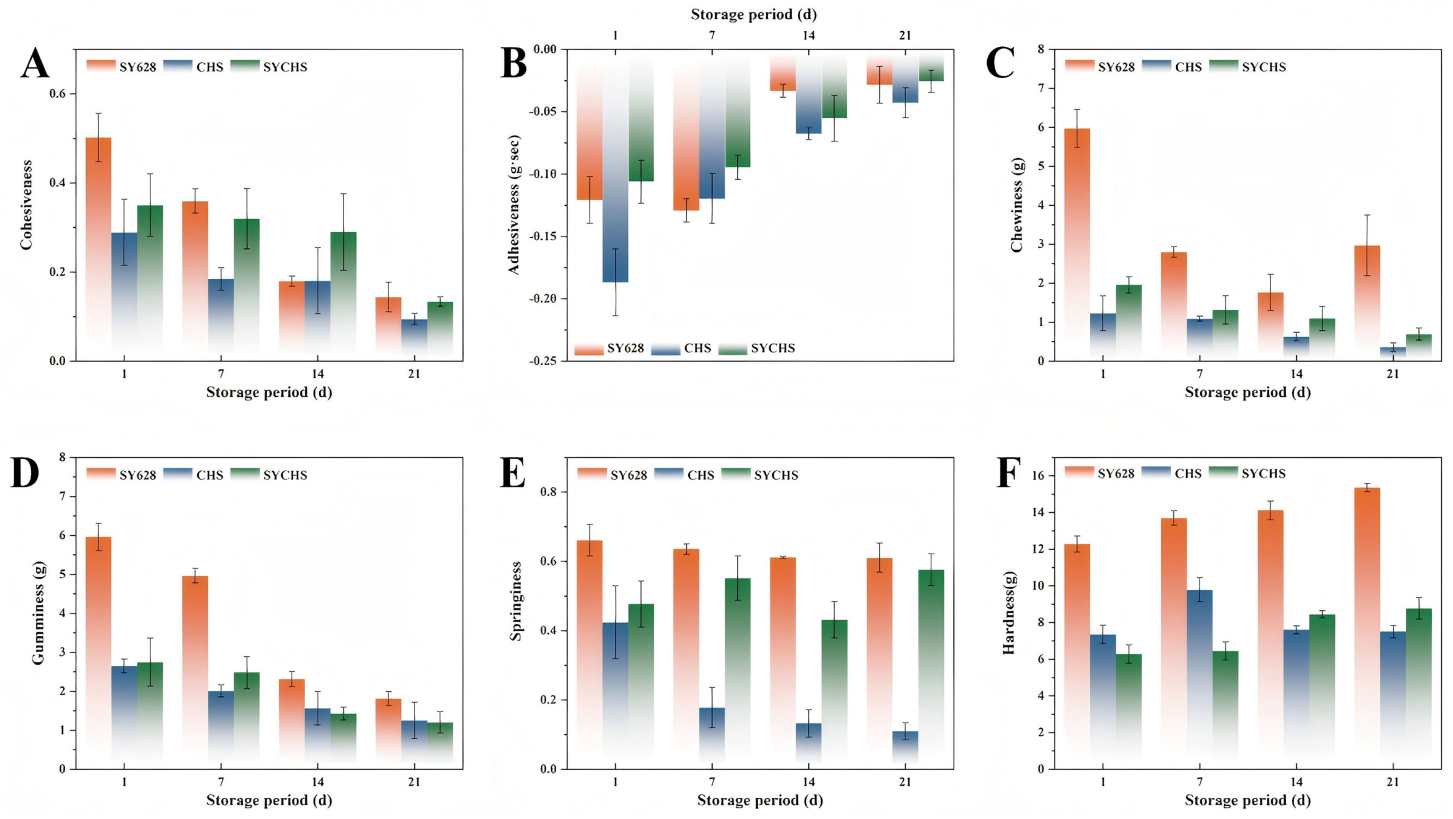
Texture analysis: **(A)** cohesiveness, **(B)** viscosity, **(C)** elasticity, **(D)** adhesiveness, **(E)** hardness, **(F)** chewiness.

Texture properties constitute a significant component of the sensory quality of foods, exerting a direct influence on consumer acceptance and the competitiveness of products in the marketplace. As illustrated in Figure 3, a comparative analysis of the textural properties of three distinct types of soy yogurt was conducted. A comparative analysis of the samples revealed significant differences (*p* < 0.05) in the effects of different starters on the texture properties of soy yogurt. With respect to the shifting trend in cohesiveness (see Figure 3A), the SYCHS group exhibited markedly enhanced cohesiveness, exhibiting values that surpassed those of the SY628 and CHS groups across all storage periods. The augmented cohesion observed in the SYCHS group may be attributed to the synergistic fermentation of *W. confusa* and the commercial strain. This synergy fosters the structural stability of the soy yogurt by facilitating the formation of a more compact colloidal network structure (Ahaotu et al., 2017). As a result, a relatively good gel structure is maintained during storage. The elevated viscosity suggests that the soy yogurt produced by the SYCHS group yielded a greater quantity of colloidal substances during the fermentation process. This alteration in viscosity is attributable to the fermentation metabolites of lactic acid bacteria. It is plausible that SYCHS produced a comparatively substantial amount of exopolysaccharides, which contributed to the observed increase in viscosity (Ren et al., 2025).

With regard to the parameters of elasticity and cohesiveness (see Figures 3C,D), the soy yogurt from the SYCHS group demonstrated significantly higher levels of elasticity and cohesiveness. These factors are known to play a pivotal role in determining the chewiness of food. Higher elasticity and cohesiveness generally indicate that a food can maintain a more optimal structural perception and enhanced taste adaptability within the oral environment (Huang et al., 2024). The SY628 and CHS groups demonstrated reduced elasticity and cohesiveness, which could have influenced their palatability during mastication. These characteristics were consistent with their diminished cohesiveness and viscosity. A comparison of the effects of the three starter cultures on hardness and chewiness (Figures 3E,F) revealed that the SYCHS group exhibited lower hardness and better chewiness. These findings suggest that the soy yogurt consumed by the SYCHS group is perceived as having a more pleasant taste and provides a relatively soft and moderate chewing experience. Conversely, the SY628 and CHS groups, characterized by higher hardness, may impart a comparatively rough taste sensation, thereby influencing consumers overall eating experience (Zhang J. et al., 2023; Zhang X. et al., 2023).

### 3.5 Rheology analysis

**Figure 4:**
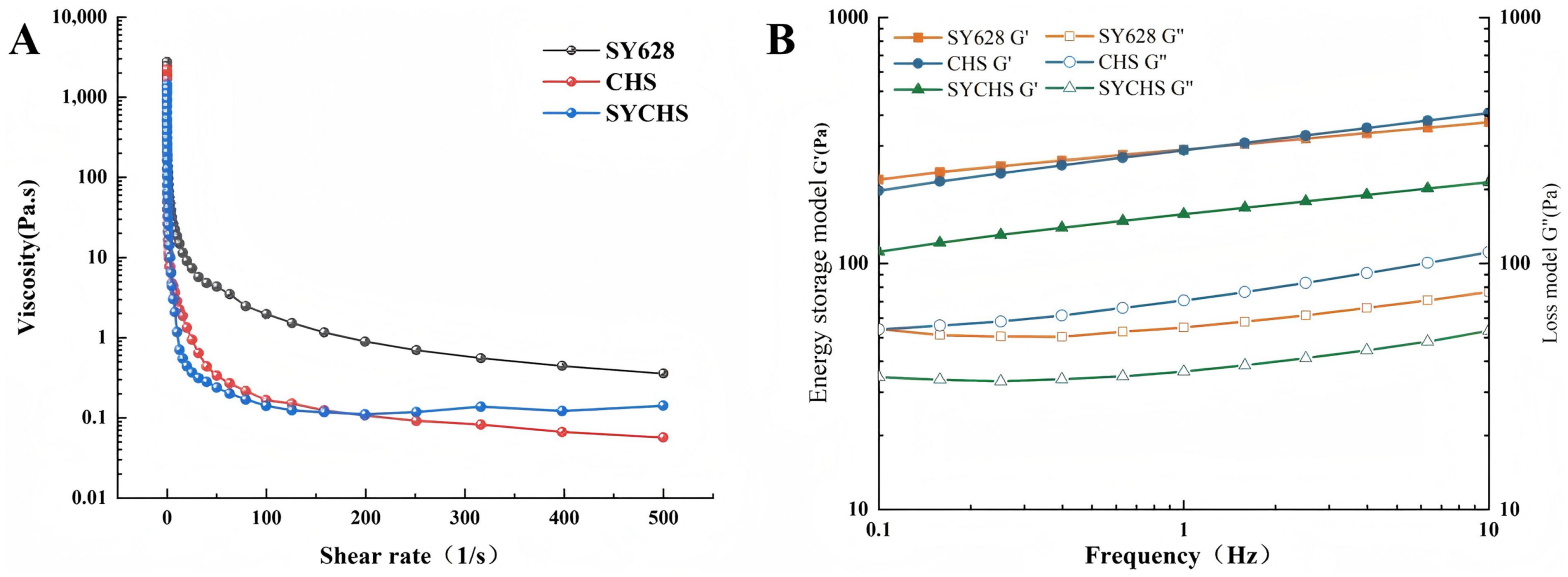
Rheological analysis: **(A)** Curve of shear rate versus viscosity; **(B)** Relationship between storage modulus and loss modulus with respect to frequency.

As illustrated in Figure 4A, all three treatments demonstrated characteristic shear-thinning behavior, characterized by a decrease in viscosity with increasing shear rate (Sun et al., 2022). The initial viscosity (at low shear rate) followed the order SY628 > CHS > SYCHS, while the viscosities of the three gradually approached each other at high shear rates. The phenomenon of shear-thinning, typically attributed to the reorganization of molecular structures within the system, suggests that the internal network architectures of the three distinct soy yogurt types were disrupted under the action of shear forces. The elevated initial viscosity of SY628 may be attributable to the comparatively elevated content of exopolysaccharides produced by its lactic acid bacteria or the tight binding of proteins in the soymilk matrix. However, the network of SY628 exhibits poor stability at high shear rates, resulting in a rapid decrease in viscosity. In contrast, SYCHS exhibits the lowest initial viscosity, likely attributable to its protein-polysaccharide network, which is characterized by increased flexibility or looseness. This network facilitates enhanced adaptability at elevated shear rates, as previously reported in the literature (Li et al., 2021).

As demonstrated in Figure 4B, for all samples, both the storage modulus (G’) and the loss modulus (G”) exhibit an increase with increasing frequency. For the storage modulus (G’), the sequence is as follows: SYCHS > CHS > SY628, which suggests that SYCHS possesses the most robust gel network structure. In terms of the loss modulus (G”), the G” values of CHS and SYCHS are close, and both are higher than that of SY628, suggesting that CHS and SYCHS possess more of the characteristics of both elasticity and viscosity. Across the entire frequency range, the G’ of all samples exceeded G,” suggesting that the gel properties of soy yogurt prevailed, thereby manifesting the system’s predominantly solid-state characteristics. The elevated G’ value of SYCHS signifies that its internal network is more stable and compact. This observation can be attributed to the elevated production of exopolysaccharides and the synergistic effect of lactic acid bacteria, which enhance the cross-linking between proteins and polysaccharides. All treatments exhibited non-Newtonian fluid characteristics, which is consistent with the typical rheological behavior of soy yogurt (Bi et al., 2020). SYCHS demonstrated superior high-shear stability, indicating that its network structure possesses increased flexibility and adaptability. The G’ of SYCHS was found to be significantly higher than that of the other two treatments, suggesting that it possesses stronger gel strength and an elastic network structure.

### 3.6 Color difference and sensory analysis

As illustrated in Table 4, CHS demonstrated the highest brightness (L* value), with a measurement of 96.73 ± 0.61. The brightness of SYCHS and SY628 was slightly lower. The L* value is a measure of the brightness of a sample; higher L* values are indicative of greater uniformity in the distribution of proteins and light-reflection characteristics during the fermentation process. The basis for the assertion that CHS has the highest brightness might be that the fermentation strains of CHS produce fewer secondary metabolites that cause color changes (Tu et al., 2024). The a* values of all samples were found to be close to −2.9, indicating that the samples exhibited a green hue with no significant differences. The greenish tint of soy yogurt may primarily originate from the natural components of the soymilk matrix, such as residual chlorophyll in beans or the oxidation of polyphenolic compounds (Dhungana et al., 2021). The b* value of CHS (17.01 ± 1.28) is significantly higher than that of SYCHS (13.31 ± 0.98) and SY628 (14.06 ± 0.44), indicating that the CHS sample tends to be more yellow. In contrast, SYCHS exhibited the lowest b* value, suggesting that its yellow hue is comparatively weak. The enhancement of the yellow color may be related to the metabolic capacity of the fermentation strain on the soymilk matrix. The total color difference of CHS (ΔE* = 12.80 ± 1.21) is significantly higher than that of SYCHS (9.13 ± 1.02) and SY628 (9.77 ± 0.42). The ΔE* values of SYCHS and SY628 are proximate, yet SYCHS exhibits a marginally diminished value, thereby suggesting that its color manifests greater proximity to the ideal white matrix. It is noteworthy that the magnitude of the ΔE* value is directly proportional to the extent of color deviation from the original state. The heightened total color difference exhibited by CHS may be associated with its elevated b* value, indicative of yellow tendency, and its more pronounced L* value. In comparison, SYCHS exhibited the lowest total color difference, suggesting that the color change during its fermentation process is relatively insignificant and that the fermentation strain exerts a more moderate influence on the color of the matrix (Zhu et al., 2024).

**Table 4:** Color difference analysis of different samples.

Color	CHS	SYCHS	SY628
L*	96.73 ± 0.61^a^	95.49 ± 1.09^ab^	94.80 ± 0.57^b^
a*	−2.93 ± 0.26^a^	−2.99 ± 0.84^a^	−2.95 ± 0.02^a^
b*	17.01 ± 1.28^a^	13.31 ± 0.98^b^	14.06 ± 0.44^b^
ΔE*	12.80 ± 1.21^a^	9.13 ± 1.02^b^	9.77 ± 0.42^b^

The * is a specific markup to indicate that the parameter is a coordinate value in a uniform color space. Different lowercase letters after peer data indicate variability.

Human sensory evaluation is important in assessing consumer market acceptance, and Supplementary Table 1 presents data on the impact of fermentation strains on the sensory attributes of soy yogurt during refrigerated storage (4°C) at day 1. The results indicate that the soy yogurt from the SY628 and SYCHS groups received significantly higher scores for parameters including color, odor, texture, and appearance compared to the CHS group. Notably, the SYCHS group demonstrated superior overall consumer acceptability.

### 3.7 Microstructure analysis

**Figure 5:**
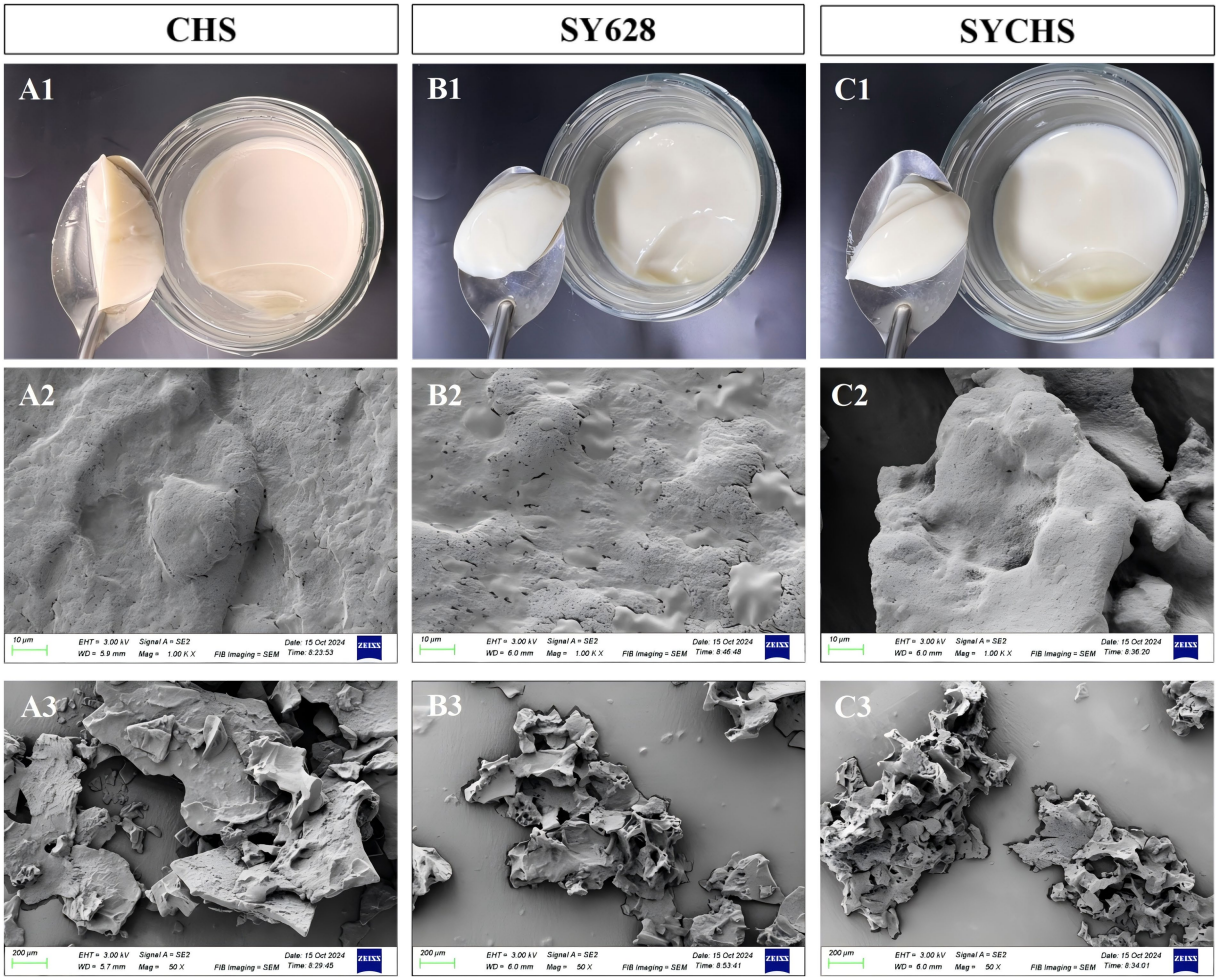
Different sample finished products and microstructures. CHS Finished Drawing **(A1)**, CHS SEM1.00k× **(A2)**, CHS SEM 50× **(A3)**; SY628 Finished Drawing **(B1)**, SY628 SEM 1.00k× **(B2)**, SY628 SEM 50× **(B3)**; SYCHS Finished Drawing **(C1)**, SYCHS SEM1.00k× **(C2)**, SYCHS SEM 50× **(C3)**.

The finished products and scanning electron microscopy (SEM) images revealed significant differences in structural characteristics and colloidal network formation of soymilk yogurt induced by different starter cultures. As shown in Figure 5A1, the CHS group exhibited a brittle texture that fractured easily under minor stress, demonstrating insufficient continuous elasticity accompanied by pronounced whey separation. At higher magnifications (Figures 5A2,A3), the CHS group displayed a smooth surface with small pores and a dense microstructure. In contrast, lower magnification observations showed a flaky surface and a homogeneous smooth cross-section. During fermentation, the CHS strain formed a compact protein-polysaccharide network, which enhanced gel strength but simultaneously restricted water migration, increased hardness, and reduced elasticity. These phenomena aligned with its elevated hardness values and adversely affected rheological properties (Luo et al., 2023). As demonstrated in Figure 5B1, the SY628 group exhibited a relatively stable texture with high elasticity and toughness, accompanied by an absence of whey separation. Upon higher magnification (Figures 5B2,B3), the SY628 group displayed a rough surface with numerous pores and cracks, along with a loose microstructure. At lower magnification, its surface appeared fragmented and irregular. The SY628 strain formed a loosely arranged and porous protein-polysaccharide network, a structure that was weakly bonded, facilitating water loss and phase separation, as evidenced by the low viscosity and storage modulus values observed in rheological analyses (Ryu and McClements, 2024).

In Figure 5C1, the SYCHS group demonstrated a non-adhesive texture (no adherence to the cup wall or spoon), significant elastic properties, and an absence of whey separation. Microscopic analysis of Figures 5C2,C3 revealed a smooth surface with moderate pore size and uniform microstructure in the SYCHS group. At lower magnification, it displayed a porous mesh-like architecture. The SYCHS strain developed a homogeneous and porous network, enabling balanced distribution of moisture, water-holding capacity, and metabolites, thereby enhancing elasticity. The rheological and textural properties of the SYCHS strain, which include a high storage modulus, moderate hardness, and superior elasticity, are attributed to its optimized microstructure (Lv et al., 2023). In summary, the soymilk yogurt from the SYCHS group exhibited a more uniform and dense gel network, demonstrating significant advantages in both texture and stability. Although the SY628 and CHS groups showed structural strengths (e.g., the porous framework of SY628 and the compact protein-polysaccharide matrix of CHS), their colloidal network stability was comparatively poor, leading to textural changes during storage. This discrepancy may be attributable to synergistic effects among microbial strains and interactions of metabolites (e.g., lactic acid and exopolysaccharides) during fermentation. Previous studies have demonstrated that exopolysaccharides (EPS) produced by *Weissella confusa* can significantly enhance the viscosity of plant-based yogurts through the formation of a three-dimensional network gel structure (Huang et al., 2025).

### 3.8 Amino acid analysis

**Figure 6:**
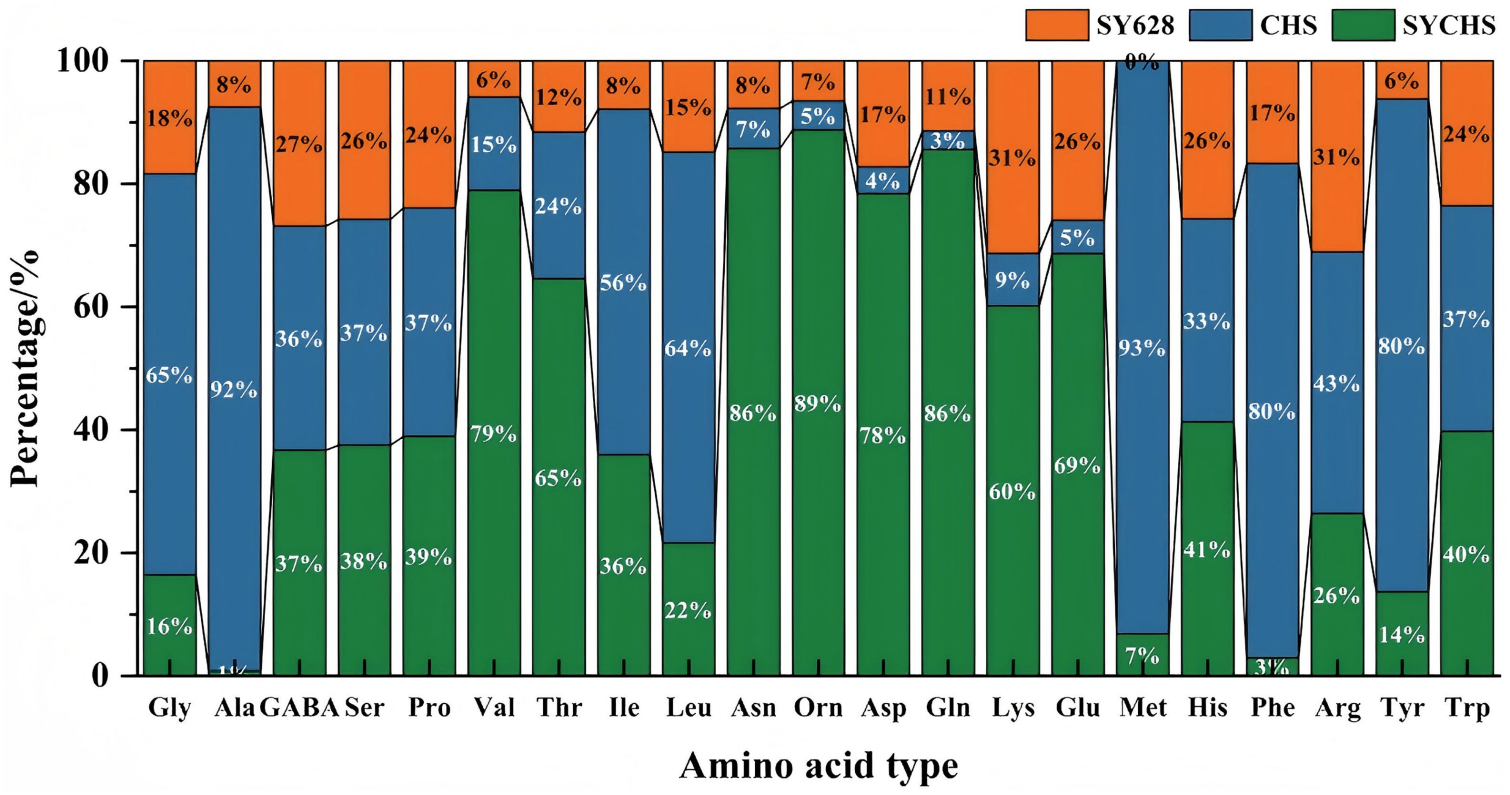
Comparison of amino acids.

As indicated in Table 5, the SYCHS group exhibited the highest total amino acid content (308.57 μg/g), which was significantly higher than that of the CHS group (208.53 μg/g) and the SY628 group (165.07 μg/g). This finding suggests that the SYCHS strain exhibits a superior capacity for protein hydrolysis and amino acid metabolism during the fermentation process (Li C. C. et al., 2024; Li S. et al., 2024). The total amino acid content of CHS was found to be higher than that of SY628, suggesting that the CHS strain may possess specific metabolic advantages. The proportion of umami amino acids in SYCHS was the highest (22.95%), and the proportions of Glu and Asp were significantly higher than those in the other two groups (the green part in Figure 6 was dominant). These amino acids have been demonstrated to play a pivotal role in enhancing the umami taste and overall flavor of soy yogurt, which aligns with prior research findings (Chen et al., 2023). In contrast, the proportion of umami amino acids in CHS (2.25%) was significantly lower than that in SY628 (12.14%), suggesting that SY628 outperforms CHS in terms of flavor enhancement. The highest proportion of sweet amino acids was observed in CHS (7.30%), particularly with significant concentrations of Ala and Ser. The proportions of sweet amino acids in SYCHS and SY628 were relatively close (3.60 and 3.43%), but SYCHS had a stronger overall amino acid metabolism capacity, resulting in a higher absolute content of sweet amino acids. The proportion of bitter amino acids in CHS was the highest (7.40%), indicating that this strain might release more amino acids related to bitterness during the fermentation process. SY628 exhibited the second-highest proportion of bitter amino acids (4.59%), which might contribute to a moderate bitter taste. In contrast, SYCHS exhibited the lowest proportion (3.35%), suggesting that the SYCHS strain produced a comparatively lower content of bitter substances. The content of essential amino acids in SYCHS was the highest (43.34 μg/g), indicating that the soy yogurt fermented by this strain better meets the requirements of nutritional fortification. In comparison, the content of essential amino acids in CHS (40.21 μg/g) surpassed that of SY628 (23.95 μg/g), indicating that CHS retains its capacity to ensure adequate nutrition (Ng et al., 2024). SYCHS exhibited a predominant presence of amino acids, particularly umami amino acids such as Glu, Asp., and Gln, along with essential amino acids including Thr, Val, and Leu, characterized by a balanced distribution of overall proportions. The mixed fermentation culture SYCHS contains *Bifidobacterium bifidum*, which produces the enzyme threonine aldolase. This enzyme catalyzes the conversion of threonine into acetaldehyde, which is an important flavor compound in yogurt (Ott et al., 2000).

**Table 5:** Amino acid composition of different samples.

Amino acid	SYCHS	CHS	SY628
Total amino acid (μg/g)	308.57 ± 4.33^a^	208.53 ± 2.14^b^	165.07 ± 3.51^c^
Delicious amino acids (%)	22.95 ± 1.17^a^	2.25 ± 0.19^c^	12.14 ± 0.35^b^
Sweet amino acids (%)	3.60 ± 0.35^b^	7.30 ± 0.43^a^	3.43 ± 0.15^b^
Bitter amino acid (%)	3.35 ± 0.17^c^	7.40 ± 1.00^a^	4.59 ± 0.31^b^
Essential amino acid (μg/g)	43.34 ± 1.86^a^	40.21 ± 2.66^b^	23.95 ± 1.49^c^

Different lowercase letters after peer data indicate variability.

### 3.9 Fatty acid analysis

As indicated in Table 6, the SYCHS strain exhibited the highest total fatty acid content (1818.95 μg/g), which was significantly higher than that of the SY628 (1643.32 μg/g) and CHS (1177.01 μg/g) strains. This observation suggests that during the fermentation process, the SYCHS strain may promote lipid metabolism or the retention of residual fat more effectively. Conversely, CHS exhibited the lowest total fatty acid content, which could be attributed to the relatively lower metabolic capacity of the strain or its less efficient substrate utilization. The SYCHS strain demonstrated the highest content of saturated fatty acids (387.70 μg/g), with C16:0 (palmitic acid) and C18:0 (stearic acid) being the primary contributors. Saturated fatty acids play a role in maintaining the product’s texture; however, elevated levels may hurt its health benefits (Wongsurawat et al., 2023). SY628 exhibited the second-highest content (353.81 μg/g), suggesting that it retained a certain amount of saturated fatty acids during the process of fatty acid metabolism. In contrast, CHS exhibited the lowest content (249.61 μg/g), suggesting a reduced generation of saturated fatty acids during the fermentation process. The SYCHS sample exhibited the highest content of monounsaturated fatty acids (409.75 μg/g), which is likely to consist primarily of C18:1n9c (oleic acid), among other components. The presence of monounsaturated fatty acids has been linked to the health benefits of the product and can serve as a potential source of anti-inflammatory and cardiovascular protective factors (Li H. et al., 2022; Li L. et al., 2022). Conversely, CHS exhibited the lowest content (278.29 μg/g), which could potentially lead to a marginally diminished performance of its products in terms of flavor or functionality. The content of polyunsaturated fatty acids in SYCHS was the highest (1021.16 μg/g), significantly higher than that in SY628 (920.34 μg/g) and CHS (648.03 μg/g). These polyunsaturated fatty acids encompass essential fatty acids that have been demonstrated to be beneficial to health, including C18:2n6c (linoleic acid) and C18:3n3 (*α*-linolenic acid).

**Table 6:** Fatty acid composition of different samples.

Fatty acid	SYCHS	CHS	SY628
Total fatty acids (μg/g)	1818.95 ± 3.15^a^	1177.01 ± 5.97^c^	1643.32 ± 6.64^b^
Saturated fat (μg/g)	387.70 ± 3.37^a^	249.61 ± 0.65^c^	353.81 ± 3.69^b^
Monounsaturated fatty acid (μg/g)	409.75 ± 1.83^a^	278.29 ± 4.49^c^	368.73 ± 1.16^b^
Polyunsaturated fatty acid (μg/g)	1021.16 ± 2.47^a^	648.03 ± 5.78^c^	920.34 ± 190^b^

Different lowercase letters after peer data indicate variability.

**Figure 7:**
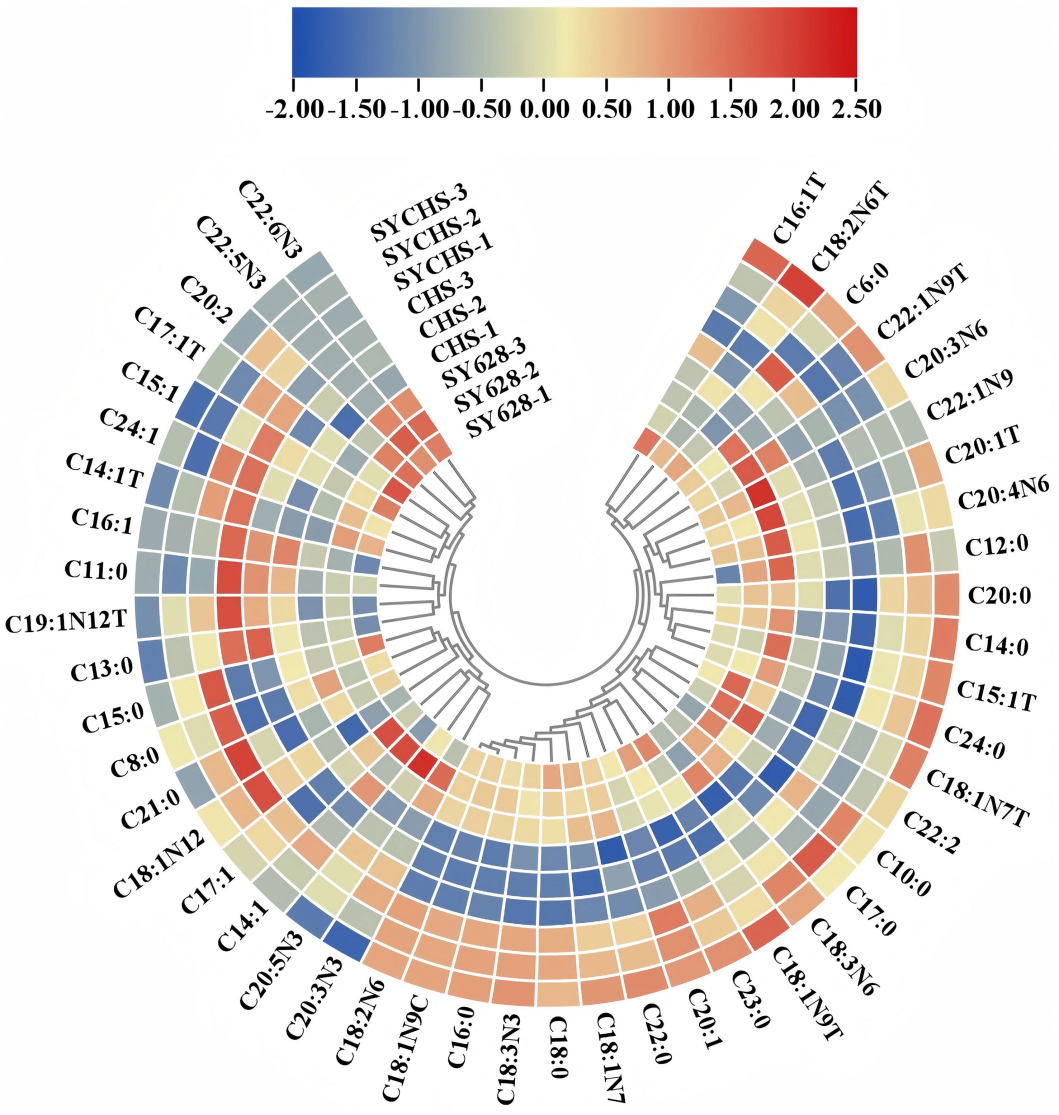
Heat map and cluster analysis of fatty acids.

As illustrated in Figure 7, a comprehensive analysis of fatty acid composition reveals notable disparities among diverse starter cultures. For SYCHS (outer ring with the highest red proportion), the data demonstrate dominance in various fatty acids, such as C18:2n6c (linoleic acid) and C18:3n3 (α-linolenic acid), and a marked contribution to PUFA (polyunsaturated fatty acids). The relatively high proportions of C16:0 and C18:1n9c in SYCHS also demonstrate its advantage in balancing SFA (saturated fatty acids) and MUFA (monounsaturated fatty acids). For SY628 (outer ring with the second-highest red proportion), the performance in C16:1 and C18:1n9c is notable, suggesting a substantial contribution of SY628 to monounsaturated fatty acids. It closely resembles SYCHS in certain PUFAs, including C20:4n6 (arachidonic acid). In contrast, CHS (outer ring dominated by blue) exhibits comparatively lower contents of most fatty acids, particularly crucial PUFAs such as C18:2n6c and C18:3n3. The relatively low proportion of saturated fatty acids may result in weaker lipid-metabolism-related properties of its products (Gao et al., 2020).

### 3.10 Flavor omics analysis

#### 3.10.1 Statistics on quantities of identified flavor substances

**Figure 8:**
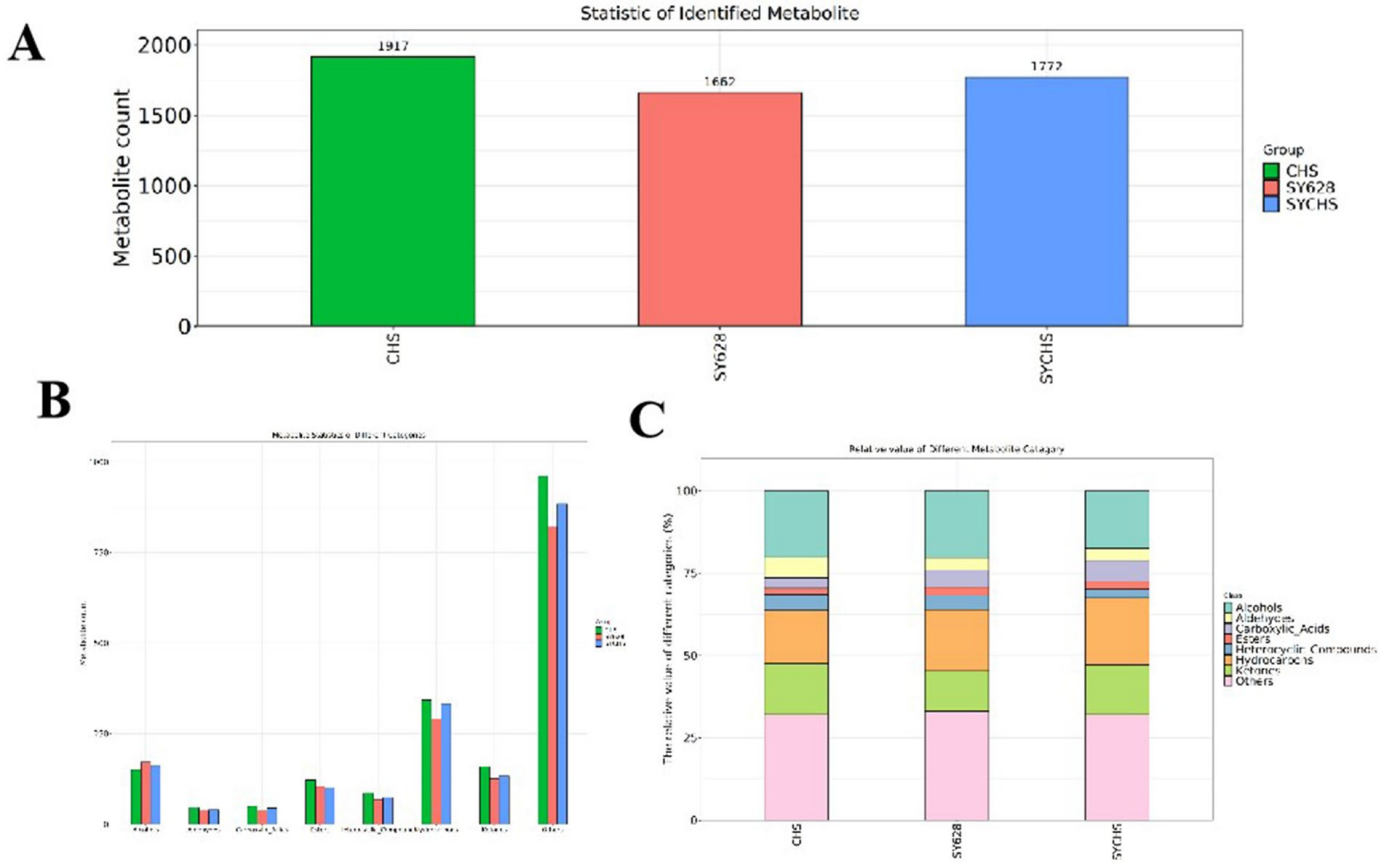
**(A)** Statistics of identified compounds **(B)** Statistics of different classes of metabolites **(C)** Stacked bar chart.

The raw data of flavor compounds were analyzed using the chroot—maTOF software and sensory annotations were retrieved from the NIST2020 database (He and Jeleń, 2024). After the elimination of extraneous data, the identification information of the flavor compounds was obtained. As illustrated in Figure 8A, the total number of compounds identified in the samples of each species is displayed. The highest number of identified flavor compounds was found in CHS, followed by SYCHS, and the lowest number was detected in SY628. Figure 8B illustrates the number of flavor compounds corresponding to each category. This figure provides summary statistics of the main categories, including hydrocarbons, aldehydes, esters, acids, ketones, alcohols, ethers, phenols, and heterocyclic compounds. These volatile flavor substances are present in all flavor compounds. Among the species analyzed, SYCHS contained the largest amounts of Carboxylic Acids and Hydrocarbons; CHS had the highest contents of Aldehydes, Heterocyclic Compounds, and Ketones; while SY628 had the highest contents of Alcohols and Esters, showing a distinct difference from other species. The flavor of soy yogurt consists of two distinct components: identifiable taste and odor characteristics, as well as inseparable complex characteristics. The Flavor DB website was utilized to systematically analyze and compare the sensory flavors of compounds in diverse samples (Sun et al., 2023). Figure 8C illustrates the relative contents of flavor compounds in soymilk yogurt treated with different starter cultures through a compositional stacking plot. As shown, the CHS group exhibited higher levels of aldehydes, heterocyclic compounds, and ketones, contributing to its distinct flavor profile. The SY628 group demonstrated dominance in alcohols and esters, which may underpin its more pronounced aroma profile. In contrast, the SYCHS group displayed a balanced distribution of flavor compounds, with particularly elevated levels of acids and hydrocarbons, indicating a more complex and harmonious flavor composition. These differences in flavor compound composition reflect variations in metabolic pathways during fermentation with respective starter cultures, thereby driving significant flavor differentiation among the yogurt variants. Such diversity not only enriches the spectrum of flavor characteristics in soymilk yogurt but also expands its potential for diversified market applications and consumer preferences (Liu M. et al., 2024; Liu Y. et al., 2024).

#### 3.10.2 Multivariate statistical analysis

**Figure 9:**
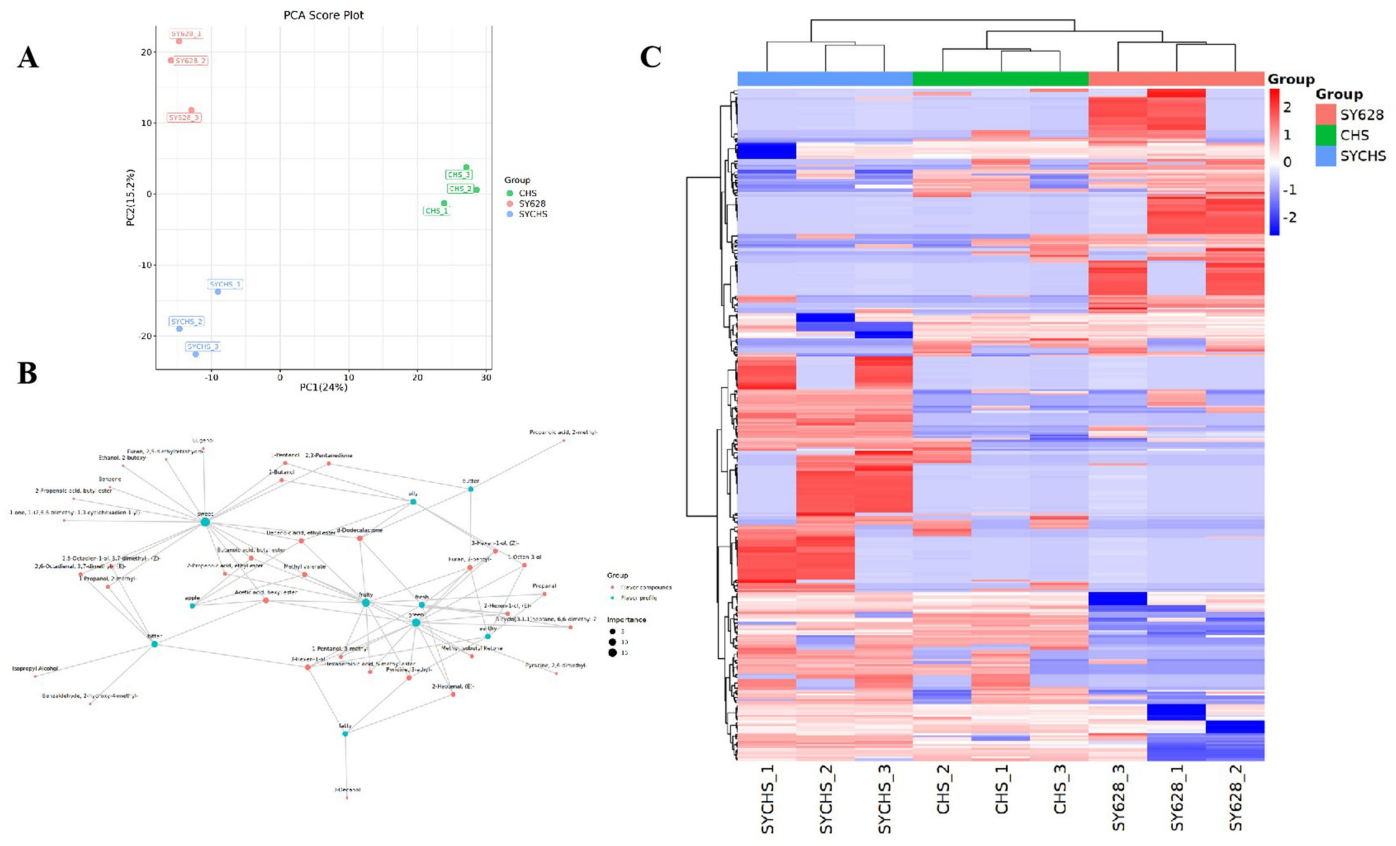
**(A)** PCA score plot **(B)** Hierarchical clustering heat map of differential compounds **(C)** Sensory flavor profile and flavor compound network.

Principal component analysis (PCA) is a detection method based on multivariate statistics. It utilizes the signal intensity of flavor substances to highlight the differences among samples (Gu et al., 2023). To investigate the impact of metabolites in the three samples, PCA analysis was conducted on the data. In this experiment, before performing multivariate statistical analysis, the data were subjected to Par (Pareto scaling) transformation to obtain more reliable and intuitive results. The PCA model was not overfitted (Figure 9A), thus enabling a global and descriptive assessment of the sample distribution to identify natural groupings, trends, and outliers. As illustrated in Figure 9A, PC1 and PC2 accounted for 24 and 15.2%, respectively, with a cumulative variance contribution rate reaching 39.2%. This finding suggests that these two principal components can, to a certain extent, reflect the differences among the samples. The results of the principal component analysis demonstrate that the distances between the three types of samples are relatively pronounced, suggesting that the disparities in their qualities are substantial. The nature of this differentiation is driven by key flavor compounds, with some samples being the dominant variable in PCA clustering due to the dominance of the content of aldehydes (conferring a grassy aroma), esters (building a fruity base), or ketones (providing a creamy aroma). This phenomenon may be attributed to the influence of the metabolic properties of different strains on the production of flavor compounds. As previously noted by Kazimírová and Rebroš (2021), the microbial regulation of aldehyde and other compound synthesis within the fermentation system exerts a direct influence on the flavor differentiation of the samples. This finding provides a theoretical foundation for the grouping characteristics presented by the PCA, thereby systematically elucidating the underlying causes of flavor variations in the samples.

#### 3.10.3 Differential compound and flavor characterization

To identify differential organic compounds, we employed a t-test with a *p*-value less than 0.05 and a VIP value greater than 1. The identification of 371 differential organic compounds was achieved, and their relative contents were plotted as a heatmap to demonstrate the differences among species. As illustrated in Figure 9B, the contents of these compounds in SYCHS were found to be significantly higher compared to other samples, while the contents in CHS were found to be significantly lower compared to other samples. This finding aligns with the PCA results, suggesting that these compounds play a pivotal role in the variation in flavor among species. To further elucidate the aforementioned findings, the present study constructed a metabolism-flavor association network diagram (Figure 9C) using the Igraph network analysis tool and the Flavordb flavor compound database (Goel et al., 2024). This network diagram depicted the relationship between flavor compounds and their sensory characteristics. In the network diagram, red nodes represent flavor compounds, and green nodes represent flavor characteristics. The lines connecting these nodes reflect the strength and direction of the correlations between them. Through the analysis of the size of the nodes and the distribution of the connecting lines in the diagram, it is possible to visually identify the compounds that contribute the most to specific flavor characteristics. Among the compounds identified in the network diagram, those associated with sweetness, such as Eugenol and Furan, exhibit relatively large nodes, suggesting their significance in the perception of sweetness. Conversely, compounds associated with bitterness, such as isopropyl alcohol and benzaldehyde, exhibit comparatively diminutive nodes in the network diagram. Nevertheless, their contributions to bitterness are not negligible. Furthermore, the formation of fruity aroma characteristics is significantly correlated with compounds such as acetic acid, hexyl ester, and 2-propenoic acid, ethyl ester, demonstrating the crucial role of these compounds in terms of aroma (Lan et al., 2024). The potential interactions among flavor components may ultimately lead to the overall flavor differences in different samples.

#### 3.10.4 Analysis of key aroma presenting substances in different samples

**Figure 10:**
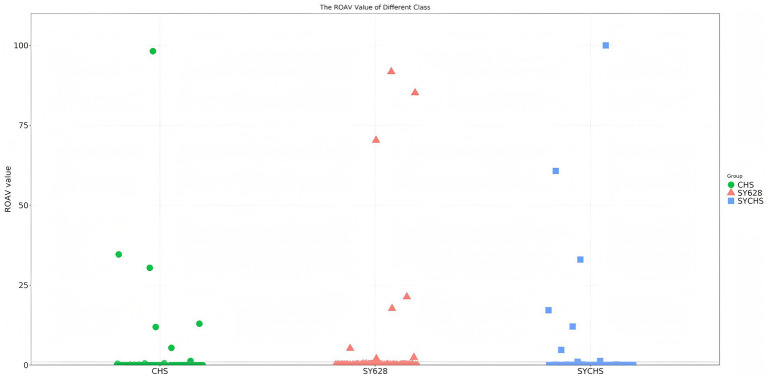
Volatile-flavor substances scatter plot of ROAV.

ROAV is employed to elucidate the contribution of each aroma compound to the overall aroma characteristics of a given sample. Typically, when ROAV ≥ 1, it signifies that the compound exerts a direct influence on the flavor profile of the sample (Wang et al., 2021) As demonstrated in Figure 10, the key aroma-contributing components (ROAV > 1) of the three samples exhibit significant disparities. To further analyze the contribution degree (ROAV) of different volatile substances to the aroma, a total of eight key flavor odor-active substances with ROAV ≥ 1 were screened out from the volatile substances of the samples (Table 7). Among them, the substance 2,3-butanedione typically imparts a creamy, buttery, or yogurt-like odor (Wang et al., 2024). In SYCHS, its ROVA reaches a remarkable 100.00, potentially contributing to a rich, creamy, or yogurt-like flavor profile. Conversely, in CHS, the ROVA is only 4.63, indicating a comparatively diminished creamy flavor.

**Table 7:** ROAV values of main aroma active components of different samples.

Compound	Odor threshold	Chemical equation	Odor character	ROAV		
				SY628	CHS	SYCHS
2-Nonenal, (E)-	0.0002	C_9_H_16_O	Fatty, Cucumber	91.77	98.20	60.74
2-Octenal, (E)-	0.003	C_8_H_14_O	Nuts, Green, Fatty	17.73	12.95	12.08
Heptanal	0.003	C_7_H_14_O	Citrus, Fatty, Rancid	5.22	5.40	4.76
1-Octen-3-one	0.005	C_8_H_14_O	Mushroom-Like	21.38	11.92	17.17
2,3-Butanedione	0.002	C_4_H_6_O_2_	pleasant, buttery	85.15	34.63	100.00
Butanoic acid, 3-methyl-	0.02	C_5_H_10_O_2_	Rancid Cheese, Sweaty, Putrid	2.43	<1	1.04
Furan, 2-pentyl-	0.006	C_9_H_14_O	Green Beans, Vegetable	70.31	30.43	33.01
Acetic acid	0.4	C_2_H_4_O_2_	Pungent, Vinegar	2.03	<1	1.27

### 3.11 Multivariate analysis

**Figure 11:**
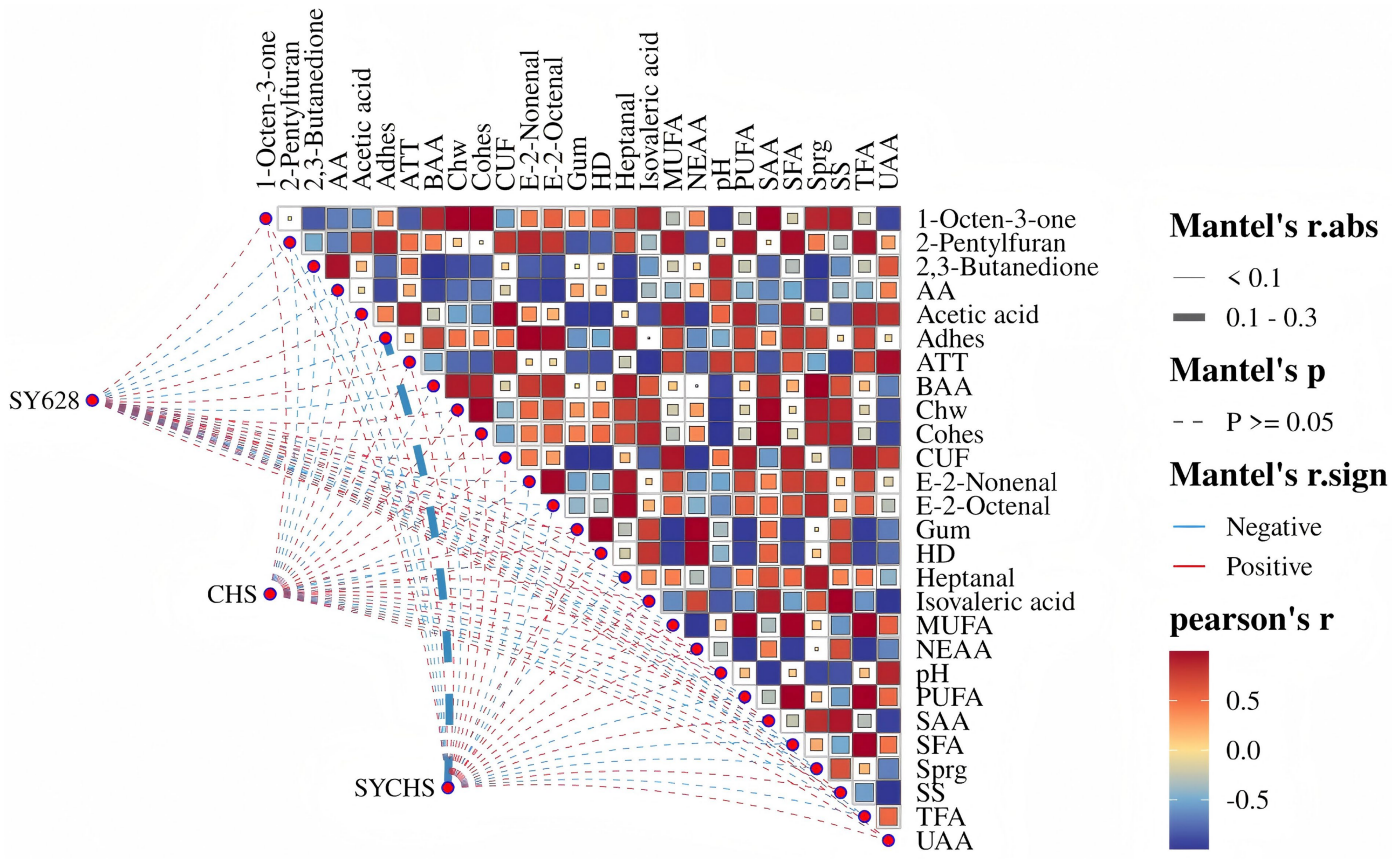
Multivariate correlation network visualization based on mantel test and Pearson correlation.

As illustrated in Figure 11, the impact of distinct starter cultures on the composition of soy yogurt compounds is demonstrated, incorporating the findings of both Pearson correlation analysis and the Mantel test. The color of the squares in the figure denotes the strength and direction of the correlation between compounds. A red square indicates a positive correlation, while a blue square indicates a negative correlation. The color bar represents the magnitude of the correlation coefficient. The observed patterns in the squares are indicative of the statistical significance of the Mantel test. Solid squares indicate statistically significant correlations (*p* < 0.1), striped squares indicate correlations between 0.1 and 0.3, and blank squares indicate non-significant correlations (*p* ≥ 0.05) (Zhou et al., 2024). Furthermore, the color and thickness of the lines connecting different treatments represent the direction and strength of the correlation in the Mantel test. Red lines signify positive correlations, while blue lines denote negative correlations. The thickness of the lines is indicative of the strength of the correlation.

As illustrated by the network heatmap, the SYCHS samples exhibit substantial correlations with numerous indicators. The key characteristics of the SYCHS samples, to a certain extent, elucidate the superior performance of the samples in terms of flavor and quality. An examination of Mantel’s r.abs and r.sign analyses reveals a relatively strong positive correlation between SYCHS and volatile flavor substances, including 1—Octen—3—one, 2,3—Butanedione, and others. Additionally, a correlation is observed between SYCHS and quality indicators such as adhesiveness, hardness, and chewiness. This finding suggests that SYCHS may enhance the sensory experience and quality stability of soy yogurt by significantly improving these characteristics. Conversely, the negative correlation factors that are significantly associated with SYCHS (e.g., certain fatty acids) may imply its potential to inhibit the formation of some undesirable flavors during the lipid metabolism process. In summary, SYCHS integrates the advantages of *W. confusa* SY628 and commercial starter cultures, demonstrating a significant synergistic effect in flavor complexity and texture improvement, providing a mechanistic basis for the quality enhancement of soy yogurt. These results suggest that SYCHS possesses not only distinctive advantages in enhancing flavor and texture but also offers novel insights for the functional development of soy-based fermented products.

## 4 Conclusion

This study systematically evaluated the safety of the *W. confusa* SY628 and its application effect in soy yogurt fermentation. The safety experiments yielded results indicating that the *W. confusa* SY628 exhibited excellent safety characteristics, including non-hemolytic activity and sensitivity to a series of antibiotics. Its remarkable tolerance to acid and bile salts further substantiates its probiotic potential.

The fermentation of soy yogurt was carried out using three distinct approaches: the *W. confusa* SY628, commercial bacterial powder CHS, and their combined starter SYCHS. The physical, and chemical properties and flavor quality of the yogurt were measured. These results demonstrated that during the storage period, the SYCHS group exhibited superior fermentation performance. The pH value of the SYCHS group was found to be moderate, and its total acid content was found to be significantly higher compared to the other groups. Furthermore, the viable cell count remained at a relatively high level, indicating its excellent proliferation ability and adaptability. In comparison to the other two starters (SY628 and CHS), SYCHS demonstrated superior performance in enhancing the textural characteristics of soy yogurt. Furthermore, SYCHS exhibited minimal alterations during storage. This phenomenon can be attributed to the synergistic effect between *W. confusa* and commercial bacteria. The enhanced production of exopolysaccharides by SYCHS has been demonstrated to regulate the protein network of soy yogurt, thereby promoting texture stability and enhancing its sensory appeal.

In the rheological analysis, the SYCHS group exhibited the highest storage modulus (G’), indicating that its internal network structure was more stable, compact, and adaptable. Furthermore, the SYCHS group demonstrated lower brightness and color difference values, suggesting that it underwent less color change during fermentation and possessed a milder flavor profile. Furthermore, the SYCHS group exhibited a preponderance in total amino groups and various amino acids, particularly umami amino acids such as Glu, Asp., and Gln, in addition to essential amino acids (e.g., Thr, Val, and Leu). This finding suggests an overall balanced proportion of amino acids. The SYCHS group demonstrated a significantly higher total fatty acid content compared to other groups, particularly in essential fatty acids that are known to promote health (e.g., C18:2n6c, linoleic acid; C18:3n3, *α*-linolenic acid).

The results of flavoromics analysis revealed that the SYCHS group exhibited the most substantial number of identified key flavor substances. In addition, the ROAV values of substances such as 2,3-butanedione were found to be considerably higher in the SYCHS group compared to other groups, thereby substantiating its superior flavor profile. The results of multivariate statistical analysis indicated significant differences in the quality of the three samples, primarily concentrated in amino acids, physical and chemical indicators, and texture. The synergistic interplay among these indicators culminated in the distinctive taste and flavor profile of the samples. In summary, the synergistic fermentation of the *W. confusa* SY628 and commercial bacteria significantly enhanced the overall quality of soy yogurt. This finding provides new ideas and theoretical support for the development of plant-based foods. The results of this study established a foundation for the optimization of the fermentation process of soy yogurt and the enhancement of its market competitiveness. Consequently, this will promote the innovation and sustainable development of functional foods.

## Data Availability

The original contributions presented in the study are included in the article/Supplementary material, further inquiries can be directed to the corresponding author.
